# High-fat diet aggravates postoperative cognitive dysfunction in aged mice

**DOI:** 10.1186/s12871-018-0482-z

**Published:** 2018-02-13

**Authors:** Lan Wei, Minmin Yao, Zhimeng Zhao, Hui Jiang, Shengjin Ge

**Affiliations:** 1 Department of Anesthesia, Zhongshan Hospital Qingpu Branch affiliated to Fudan University, Shanghai, China; 2 Department of Anesthesia, Zhongshan Hospital affiliated to Fudan University, Shanghai, China

**Keywords:** High-fat diet, Postoperative cognitive dysfunction, Sirt1, Apoptosis

## Abstract

**Background:**

Silent Information Regulator 1 (Sirt1) and apoptosis play key roles in postoperative cognitive dysfunction (POCD). Consuming a high-fat diet (HFD), a prevalent type of diet in modern society, has been increasingly recognized as contributing to neurodegenerative diseases. Although Sirt1 and apoptosis are significant responders to HFD in the brain, little is known regarding the functional correlations between HFD and POCD.

**Methods:**

Thirty-two aged C57BL/6 male mice were randomly divided into 2 groups: an ad libitum (AL) group (fed a regular diet) and high-fat diet (HF) group (fed a high-fat diet). After 8 weeks, the animals were divided into four sub-groups: an ad libitum control (ALC) group, ad libitum surgery (ALS) group, high-fat diet control (HFC) group, and high-fat diet surgery (HFS) group. The ALS and HFS groups were exposed to 3% sevoflurane in 33% oxygen for 3 h and were subsequently subjected to exploratory surgery to establish the POCD model. The ALC and HFC groups were treated with 33% oxygen for 3 h without surgery. After 48 h, the learning and memory abilities of mice in each group were tested using the Morris water maze (MWM). The expression levels of Sirt1, Bcl-2, Bax and caspase-3 cleaved were detected by western blot.

**Results:**

The MWM and western blotting results showed that the learning and memory abilities were decreased in the HFC group compared with the ALC group. The learning and memory abilities and the expression of Sirt1 in the hippocampus in the HFS group were significantly decreased compared with the other groups. A significant decrease in Sirt1 expression was also observed in the HFC group compared with the ALS group. The level of Bcl-2 was lower in the HFS group than in the HFC and ALC groups. The expression levels of caspase-3 cleaved and Bax increased in the HFS group compared with the HFC group. Moreover, the expression of caspase-3 cleaved was higher in the HFC group than in the ALS group.

**Conclusion:**

HFD can aggravate POCD in aged C57BL/6 mice, an effect that may be related to the inhibition expression of Sirt1 and the promotion of neuronal apoptosis.

## Background

Western eating habits, characterized by red meat, refined sugar, refined cereals, nuts and other saturated fats, are gaining popularity in modern society. In turn, the prevalences of overweight and midlife obesity are increasing. In the typical American diet, fat provides 35% caloric per day [1]. A large number of studies have shown that a HFD is a risk factor for many diseases, such as type 2 diabetes, hypertension, gastrointestinal diseases and tumors [2–5], as well as for various forms of accelerated cognitive decline [6, 7]. Recently, much more attention has been paid to the relationship between HFD and cognitive function. It has been shown that a HFD contributes to cognition dysfunction and neurodegenerative diseases in animals that have not been operated upon surgically [8, 9]. In addition, reduced hippocampal synaptic plasticity and impaired cognitive abilities, such as decreased spatial learning and memory, have been linked to HFD in animal studies [10]. Animal studies have proven that a HFD can impair the cognitive function of normal mice, as indicated by the decreased expression of Sirt1, [11] which modulates learning and memory function by regulating the expression of amyloid-β (Aβ) [12, 13]. In addition, the activation of Sirt1 can delay the onset of neurodegeneration [14]. Clinical research has also found that consuming a large amount of saturated fatty acids is a major risk factor for neurodegenerative diseases [15]. In addition, previous studies have shown that a HFD can induce apoptosis of neurons in the hypothalamus [16] but not in the hippocampus.

Patients can experience cognitive impairment following surgery and anesthesia. POCD, characterized by learning and memory impairments, is a common complication after surgery, often occurring in elderly patients. There is no effective treatment for POCD at present. Animal studies have shown that the effects of HFD on brain inflammation, insulin resistance and brain-derived neurotrophic factor expressions can decrease the cognitive function of normal animals [17, 18]. However, the relationship between HFD and cognitive impairment after surgery or POCD is unclear. Moreover, whether HFD aggravates POCD, affects the expression levels of Sirt1 or influences hippocampal apoptosis have not been investigated. Therefore, we used a HFD model and a POCD model to assess the following: 1) whether HFD can lower the learning and memory abilities of aged mice after surgery, and 2) whether the expression of Sirt1 or apoptosis factors are altered in the hippocampus of model mice.

## Methods

### Animals and diet

The aged C57BL/6 male mice in this study were 13–14 M old, as the C57BL/6 mice in the experiment by Zhang [19] were 12 M old. The mice weighed 28–36 g and were purchased from Shanghai Silaike Company, laboratory animal license number 2008001622124. All of the animal experiments were approved by the animal ethics committee of Zhongshan Hospital, Fudan University. After acclimating to the laboratory conditions for 7 d, all of the mice were randomly divided into two groups: an ad libitum (AL) group (*n* = 16 in each group), which continued to receive a regular diet (fat content of 5.28%); and a high-fat diet (HF) group, which was fed a HFD. All of the animals were housed in individual cages with 12 h light/12 dark cycles and free access to water. The temperature and relative humidity conditions were 20–23 °C and 60%, respectively. After 8 w, the animals were divided into four sub-groups: an ad libitum control (ALC) group (*n* = 8 in each group), an ad libitum surgery (ALS) group, a high-fat diet control (HFC) group, and a high-fat diet surgery (HFS) group. The animals were weighed weekly. The HFD, composed of 74% stock diet, 10% lard compound, 7% sucrose, 5% casein, 2% fish meal, and 2% malt dextrin, was formula feed purchased from Shanghai Pu Lu Teng Biological Technology.

### Animal model

The ALS and HFS groups were placed on a heated blanket to maintain normal body temperature. A modified small animal anesthesia system (Aespire View) was used to deliver sevoflurane. The electric blanket (yuyan am-92) to maintain the temperature of the mice at about 37 °C during anesthesia. Anesthesia in the ALS and HFS groups was induced with 3% sevoflurane in 33% oxygen and maintained with 3% sevoflurane (1.5 minimal alveolar concentration, MAC) for 3 h, after which a laparotomy was performed. After shaving and cleaning the abdominal region, a 2.0 cm median abdominal incision was created. The liver, spleen, stomach, intestines and other organs were examined successively. The operation lasted for approximately 30 min. The incision was then closed with three dissolvable sutures. Multifunctional monitoring equipment (CARESCAPE Monitor B650) was used to continuously monitor the P_ET_CO_2_ and MAC of the inhalational anesthesia. A constant concentration of sevoflurane was thereby maintained. This equipment was also used to monitor SpO_2_ via pulse oximetry. Local application of an anesthetic cream (2.5% lidocaine and 2.5% prilocaine) was used to relieve postoperative pain every 8 h within 48 h after surgery. The sham groups (the ALC and HFC groups) only received 33% oxygen for 3 h. To prevent the interference of the spatial crowding effect on the subsequent behavioral tests, the original cages were used as the anesthesia chambers for the sham groups, and these groups were not monitored for SpO_2_ to avoid stress.

### Morris water maze test

Spatial learning and memory were assessed by the classic Morris water maze, which consists of an orientation navigation experiment and a spatial probe experiment. Forty-eight hours after the operation, the first portion of the test (i.e., the orientation navigation test) began, lasting for 6 d. The mice were randomly placed into the water four times a day in the four different quadrants. The swimming paths of the mice were automatically recorded by the camera system and analyzed using the corresponding software. The maximum time for mouse to search for the hidden platform was limited to 60 s, and success was defined as the mice staying on the hidden platform for 5 s. The mice were then allowed to remain on the platform for 15 s to observe the surrounding makers. To ensure the training interval for all mice was consistent, if the mouse failed to find the hidden platform within 60 s, it was guided to the platform and allowed to remain there for 20 s. For these mice, the escape latency (i.e., the time from when the mice entered the water until the hidden platform was found) was recorded as 60 s. Spatial probe tests were conducted on the 7th day. For these tests, the platform was removed, the mice were randomly placed into the water, and the swimming trajectory was recorded for 60 s. The utilized system(DigBehv-MM) automatically analyzes the selected parameters, which for this experiment were escape latency, swimming time of target quadrant, times across the platform and average speed.

### Tissue sampling

After the behavioral tests, mice were sacrificed under pentobarbital (1.5% pentobarbital sodium, 0.1 ml/ 20 g) anesthesia. The mice were then decapitated, after which the brains were extracted on ice. After separating the skin from the head, the foramen magnum was cut with scissors. Hemostatic forceps were then carefully inserted into the foramen magnum and used to break both sides of the parietal bone. The brains were removed from the cranium, and the cortex was stripped with forceps. The lower portion of the cortex, which is white and “V”-shaped in appearance, is the hippocampus. The hippocampi were dissected for biochemistry studies, snap-frozen in liquid nitrogen and stored at − 80 °C until analysis.

### Western blot

Each frozen hippocampus was cut into pieces after weighed in an EP tube. RIPA Lysis Buffer containing PMSF protease inhibitors (200 μl RIPA Lysis Buffer per 10 mg sample) was then added, and the sample was kept on the ice for 30 min. Then, the specimens were ground four times (30 s each time) in an automated sample-grinding instrument (Tissulyser-24) at 60 Hz. The samples were then centrifuged for 5 min at 4 °C degrees centigrade at 12000 rpm. The supernatant was the desired protein extract. The protein concentration was determined using the BCA method, after which loading buffer was added (1 μl loading buffer per 4 μl sample). The samples were then boiled for 5 min at 100 °C. The protein extracts were separated by 4–20%SDS-PAGE. The proteins were then transferred onto PVDF membranes. After blocking for 1 h with 10% skim milk, the membranes were washed with TBST and incubated at 4 °C overnight with primary antibodies against Sirt1, Bcl-2, Bax, caspase-3 cleaved and β-actin (all 1:1000, rabbit anti-mouse; Cell Signaling Technology, USA). These incubations were performed. The next day, horseradish peroxidase (HRP)-conjugated goat anti-rabbit (1:10,000, Hai Ming rui Biotechnology, China) was used as the secondary antibody. ECL reagents were used to detect the bands (Hai Ming rui Biotechnology, China), according to the manufacturer’s guidelines. Densitometric analyses of western blot films were performed using Image-Pro plus software.

### Statistical analysis

We chose the average of the four times a day to analysis in the MWM results. The data analysis and processing were performed using SPSS 23.0 statistical software, and all of the quantitative data are expressed as the mean ± SD. The differences among groups were compared with one-way analysis of variance (ANOVA). *P* < 0.05 was considered statistically significant.

## Results

### Changes in body weight in aged mice

**Fig. 1:**
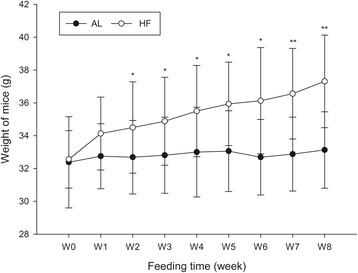
The weights of aged mice in different groups and at different time points. ^*^P < 0.05 compared with the AL group; ^**^P < 0.001 compared with the AL group

After 2 w, the body weights of the mice in the different groups began to diverge, and the weight of mice in the HF group was greater than that in the AL group (*t* = − 2.33, *P* = 0.027, Fig. 1). This difference was significant at the 8th week (*t* = − 4.57, *P* = 0.000, Fig. 1). The mice in the HF group displayed an obese phenotype. In contrast, the body weight in the AL group was only slightly changed at 8 w.

### Changes in learning and memory in aged mice

**Fig. 2:**
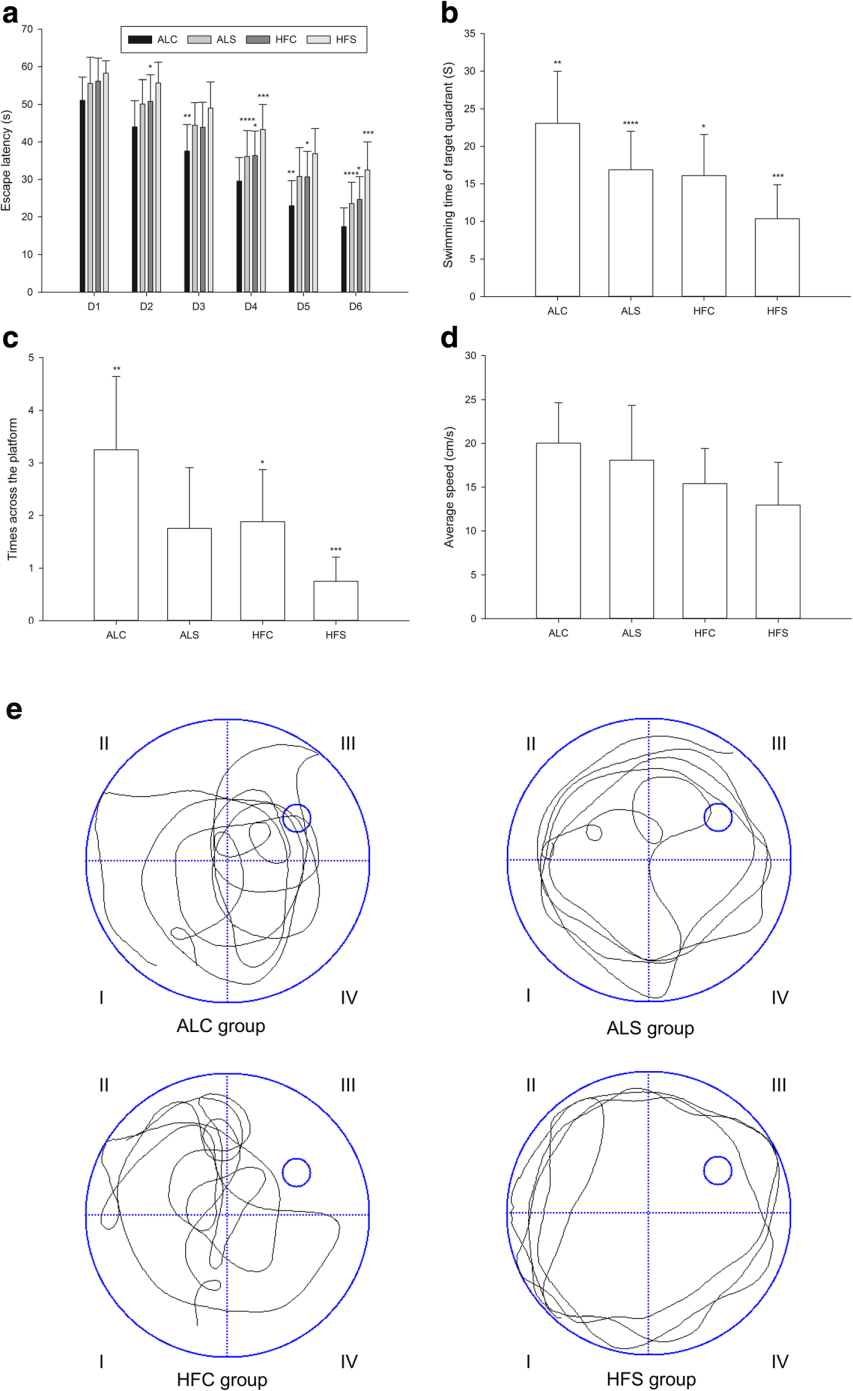
The learning and memory abilities in different groups as assessed by the Morris water maze. **a** Escape latency. **b** Swimming time of target quadrant. **c** Times across the platform. **d** Swimming speed. **e** Typical swimming patterns in the last hidden platform trial. ^*^P < 0.05 compared with the ALC group; ^**^P < 0.05 compared with the ALS group; ^***^P < 0.05 compared with the HFC group; ^****^P < 0.05 compared with the HFS group

As shown in Fig. 2a, the escape latencies were significantly longer in the ALS group (*P* < 0.05) compared with the ALC group on the 3rd and 5th day. On the 2nd, 4th, 5th and 6th day, the escape latencies in the HFC group were significantly longer than that in the ALC group (*P* < 0.05). On the 4th and 6th day, the escape latencies of mice in the HFS group were significantly longer than in the other groups (*P* < 0.05). The escape latencies in the HFC group were significantly longer. The spatial memory changes in each group are shown in Fig. 2b and c. On the 7th day, the swimming time of target quadrant was the shortest in the HFS group (*P* < 0.05, B). The times across the platform in the HFS group were lower than those in the ALC and HFC groups (*P* < 0.05, C). The times across the platform was significantly lower in the ALC group (*P* < 0.05, C) compared with the ALS group. Figure 2d shows there was no significant difference in swimming speed among the four groups.

### Changes in protein expressions in the hippocampus of aged mice

**Fig. 3:**
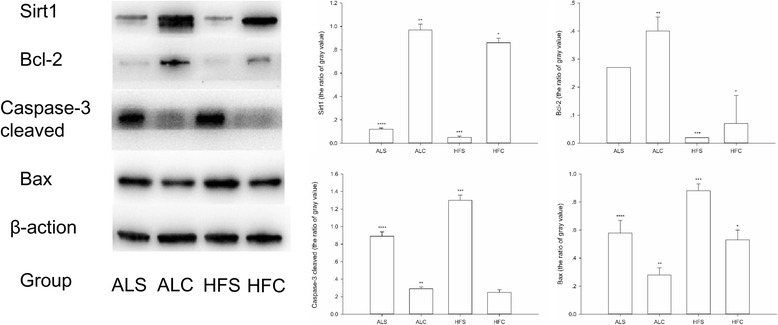
The expression levels of Sirt1, bcl-2, Bax and caspase-3 cleaved in the hippocampus tissues of different groups. A representative blot is shown at the top of the panel, and the corresponding quantitative result is shown at the bottom. ^*^P < 0.05 compared with the ALC group; ^**^P < 0.05 compared with the ALS group; ^***^P < 0.05 compared with the HFC group; ^****^P < 0.05 compared with the HFS group

As shown in Fig. 3, there were differences between each group with respect to the expression level of Sirt1 in the hippocampus. Compared with the ALC group, the level of Sirt1 was significantly lower in the HFC group (*P* < 0.05). A similar result was observed between the ALS and HFS groups (*P* < 0.05). By contrast, the Sirt1 levels were higher in the ALC group than in the ALS group (*P* < 0.05). Compared with the HFC group, the levels of Sirt1 were significantly decreased in the HFS group (*P* < 0.05), whereas expression was significantly decreased in the HFC group (*P* < 0.05) compared with the ALS group.

Compared with the ALC group, the expression of Bcl-2 significantly declined in the HFC group (*P* < 0.05) and significantly decreased in the ALS group (*P* < 0.05). The expression of Bcl-2 was lower in the HFS group than in the HFC group (*P* < 0.05). The level of caspase-3 cleaved in the HFS group was higher than in the ALS group (*P* < 0.05). The level of caspase-3 cleaved was lower in the HFC group than in the HFS group (*P* < 0.05). Compared with the ALC group, the level of caspase-3 cleaved was significantly higher in the ALS and HFC groups (*P* < 0.05). In addition, the levels of caspase-3 cleaved in the HFC group were significantly higher (*P* < 0.05) than in the ALS group. The expression of Bax was significantly different among the groups. Compared with the other groups, the level of Bax in the HFS group was the highest (*P* < 0.05). Moreover, the expression of Bax was higher in the HFC group than in the ALC group (*P* < 0.05) and higher in the ALS group than in the ALC group.

## Discussion

POCD often occurs in the early postoperative period and, in most cases, improves within a few days or a few weeks. However, this condition can persist and eventually develop into Alzheimer’s disease [20]. Although POCD can occur in all ages, it is common in patients of advanced age, and old age is the primary risk factor [20–23]. Previous studies have shown that a HFD can significantly impair learning and memory abilities in rats [24], in the absence of surgery and anesthesia. The study by Heyward [11] also suggested that normal mice fed a HFD exhibit impaired hippocampus-dependent spatial memory and a corresponding alteration in the expression of Sirt1, which has been implicated in memory consolidation. Moreover, apoptosis has been demonstrated to be a cause of many neurodegenerative diseases. In terms of POCD, previous studies have suggested that the expression levels of Bax mRNA and caspase family members are significantly elevated after surgery and the application of sevoflurane anesthesia, whereas the expression levels of Bcl-2 mRNA and Bcl-2 were significantly lowered [25, 26]. Moreover, it has been found that sevoflurane is associated with transient decreases in cognitive function [27]. Therefore, in this experiment, we established a POCD model, referred to as the Hovens mouse model [28], and investigated the expression levels of Sirt1, Bcl-2, Bax and caspase-3 cleaved. In behavioral tests, the ability of learning and memory in the HFC group decreased significantly relative to that in the ALC group. This result verified that a high-fat diet can reduce learning ability and memory in normal aged mice. Compared with the ALC group, the learning and memory abilities of the ALS group were lower. In addition, the learning and memory abilities of the HFS group were lower than in the HFC group. These results indicated that the experimental model was successfully established.

We obtained a breakthrough result in the present study. HFD not only significantly increased the weight of aged mice but also aggravated POCD. The MWM results showed that the HFS group exhibited longer escape latencies, shorter swimming times of target quadrant and fewer times across the platform than the ALS group. This result indicated that a HFD can lower the learning and memory abilities of aged mice following surgery and anesthesia. In agreement with the behavioral test, cognitive function and Sirt1 expression both decreased in the HFS group compared with the ALS group. In addition, compared with the ALC group, both cognitive function and Sirt1 expression decreased in the HFC group. Sirt1, a member of the Silent Information Regulator (SIR2) family, was originally defined as NAD+ dependent histone deacetylase. Sirt1 is now known to be involved in regulating a variety of biological processes, including the negative regulation of p53 to promote cell survival and the regulation of autophagy through its deacetylase activity. Moreover, Sirt1 protects against metabolic disease by activating Sirt1 itself and the α subunit of peroxisome proliferators-activated receptor-γ coactivator-1 (PGC-1α) and ameliorates spatial learning memory impairment induced by Aβ1–42 [29–32]. It has been proven that the absence of Sirt1 impairs cognitive abilities, including immediate memory, classical conditioning, and spatial learning. In agreement with these findings, analysis of Sirt1 knock-out mice found that these effects may be associated with defects in synaptic plasticity but not alterations in basal synaptic transmission or *N*-methyl-D-aspartate (NMDA) receptor function [32]. Moreover, studies have proven that Sirt1 regulates the corresponding function by deacetylating histones, transcription factors, and other important components of certain signaling pathways [33].

Previous research has indicated an increased activation of caspase-3, a potential apoptotic mechanism, in neurodegenerative disorders [34]. Bcl-2 and Bax are two major regulators of apoptosis, while Bcl-2 is specifically considered to be an important anti-apoptotic protein [35]. In contrast, the protein Bax can accelerate apoptosis in cells that overexpress caspase-3 [36]. Moraes [16] found that a HFD can induce neuronal apoptosis and promote the expression of the apoptosis markers Bax, caspase-3, caspase-6 and caspase-8 in the hypothalamus. In contrast, in the same model, the levels of the antiapoptotic Bcl family proteins were inhibited. In our study, the hippocampal levels of the pro-apoptosis factors Bax and caspase-3 cleaved were higher in the HFC group and HFS groups compared with the ALC and ALS groups, whereas the levels of Bcl-2 were decreased. These data suggest that a HFD likely decrease the cognitive function of mice after the operation and anesthesia by inhibiting the expression of Sirt1 and Bcl-2 and simultaneously promoting the expression of Bax and caspase-3 cleaved.

The present results differ from those of previous studies showing that normal animals fed a HFD exhibit impaired hippocampal spatial memory [11, 37–39]. In our study, we examined aged mice and investigated the impairment caused by HFD on learning and memory after surgery. In addition, using the same model, we detected changes in Sirt1 and apoptosis factors in the hippocampus. However, there are limitations of the present analysis. Specifically, although we have preliminary data on the effects of HFD on POCD and the proteins that mediate these effects in the hippocampus, we have not conclusively demonstrated that Sirt1 is the upstream regulator of apoptosis in the utilized model; this question requires further study.

## Conclusions

HFD can increase body weight, and surgery and sevoflurane anesthesia can lead to cognitive impairment in aged mice. In addition, HFD aggravates POCD, possibly by down-regulating the expression levels of Sirt1 and Bcl-2 and up-regulating the expression levels of Bax and caspase-3 cleaved, thereby increasing hippocampal neuron apoptosis.

## Abbreviations

ANOVA: Analysis of variance
Aβ: Amyloid-β
HFD: High-fat diet
MAC: Minimal alveolar concentration
MWM: Morris water maze
NMDA: Transmission or *N*-methyl-D-aspartate
PGC-1α: α subunit of peroxisome proliferators-activated receptor-γcoactivator-1
POCD: Postoperative cognitive dysfunction
SIR2: Silent Information Regulator
Sirt1: Silent Information Regulator 1
